# A further study of the spider genus *Notiocoelotes* (Araneae, Agelenidae) from Hainan Island, China

**DOI:** 10.3897/zookeys.601.7698

**Published:** 2016-06-29

**Authors:** Xiaoqing Zhang, Zhe Zhao, Guo Zheng, Shuqiang Li

**Affiliations:** 1 College of Life Sciences, Shenyang Normal University, Shenyang, Liaoning 110034, China; 2 Institute of Zoology, Chinese Academy of Sciences, Beijing 100101, China

**Keywords:** Taxonomy, Coelotinae, description, diagnosis, Southeast Asia

## Abstract

Two new *Notiocoelotes* species, *Notiocoelotes maoganensis* **sp. n.** (♂♀) and *Notiocoelotes qiongzhongensis* **sp. n.** (♂♀) are described from Hainan Island, China. In addition, the female of *Notiocoelotes membranaceus* Liu & Li, 2010 is described for the first time. DNA barcodes of three species treated in this paper were obtained for future use.

## Introduction

The spider genus *Notiocoelotes* was established by Wang et al. (2008) for one coelotine species from Hainan Island, China: *Coelotes palinitropus* Zhu & Wang, 1994. Additionally, Wang et al. (2008) described three new species: *Notiocoelotes laosensis* Wang, Xu & Li, 2008, *Notiocoelotes lingulatus* Wang, Xu & Li, 2008, *Notiocoelotes vietnamensis* Wang, Xu & Li, 2008, and transferred *Iwogumoa sparus* Dankittipakul, Chami-Kranon & Wang, 2005 to *Notiocoelotes*. Currently, eleven species of *Notiocoelotes* are known (World Spider Catalog 2016), six of which are restricted to Hainan, China. This paper provides the descriptions of two new *Notiocoelotes* species and a redescription of *Notiocoelotes membranaceus*.

## Material and methods

Specimens were examined with a LEICA M205C stereomicroscope. Images were captured with an Olympus C7070 wide zoom digital camera (7.1 megapixels) mounted on an Olympus SZX12 dissecting microscope. Epigynes and male palps were examined after dissection from the spiders’ bodies.

All measurements were obtained using a LEICA M205C stereomicroscope and are given in millimeters. Leg measurements are shown as: Total length (femur, patella + tibia, metatarsus, tarsus). Only structures (palp and legs) of the left body side were described and measured. The terminology used in the text and the figure legends follows Wang (2002). Abbreviations used in this paper and in the figure legends: A = epigynal atrium; ALE = anterior lateral eye; AME = anterior median eye; AME-ALE = distance between AME and ALE; AME-AME = distance between AME and AME; ALE-PLE = distance between ALE and PLE; CD = copulatory duct; CF = cymbial furrow; CL = conductor lamella; CO = conductor; E = embolus; EB = embolic base; ES = epigynal scape; FD = fertilization duct; LTA = lateral tibial apophysis; MA = median apophysis; PLE = posterior lateral eye; PME = posterior median eye; PME-PLE = distance between PME and PLE; PME-PME = distance between PME and PME; R = receptacle; RTA = retroventral tibial apophysis; ST = subtegulum; T = tegulum.

DNA barcodes were obtained for future use. A partial fragment of the mitochondrial gene cytochrome oxidase subunit I (COI) was amplified and sequenced for *Notiocoelotes maoganensis* sp. n., *Notiocoelotes membranaceus* and *Notiocoelotes qiongzhongensis* sp. n. using primers LCO1490-oono (5’-CWACAAAYCATARRGATATTGG-3’) (Folmer et al. 1994; Miller et al. 2010) and HCO2198-zz (5’-TAAACTTCCAGGTGACCAAAAAATCA-3’) (Folmer et al. 1994; Chen et al. 2015). For additional information on extraction, amplification, and sequencing procedures, see Zhao et al. 2013. All sequences were deposited in GenBank and the accession numbers are provided in Table 1.

**Table 1.** Voucher specimen information.

Species	GenBank accession number	Sequence length	Collection localities
*Notiocoelotes maoganensis* sp. n.	KU886075	657 bp	Baoting County, Hainan, China
*Notiocoelotes membranaceus*	KU886076	666 bp	Qiongzhong County, Hainan, China
*Notiocoelotes qiongzhongensis* sp. n.	KU886074	666 bp	Qiongzhong County, Hainan, China

All specimens (including molecular vouchers) are deposited in the Institute of Zoology, Chinese Academy of Sciences in Beijing (IZCAS).

## Taxonomy

### Family Agelenidae C.L. Koch, 1837 Subfamily Coelotinae F.O.P.-Cambridge, 1893

#### Genus Notiocoelotes

Taxon classificationAnimaliaAraneaeAgelenidae

Wang, Xu & Li, 2008

Notiocoelotes Wang et al, 2008: 11. Type species Coelotes palinitropus Zhu & Wang, 1994, from Hainan Island, China.

##### Diagnosis.

**Figure 1.**
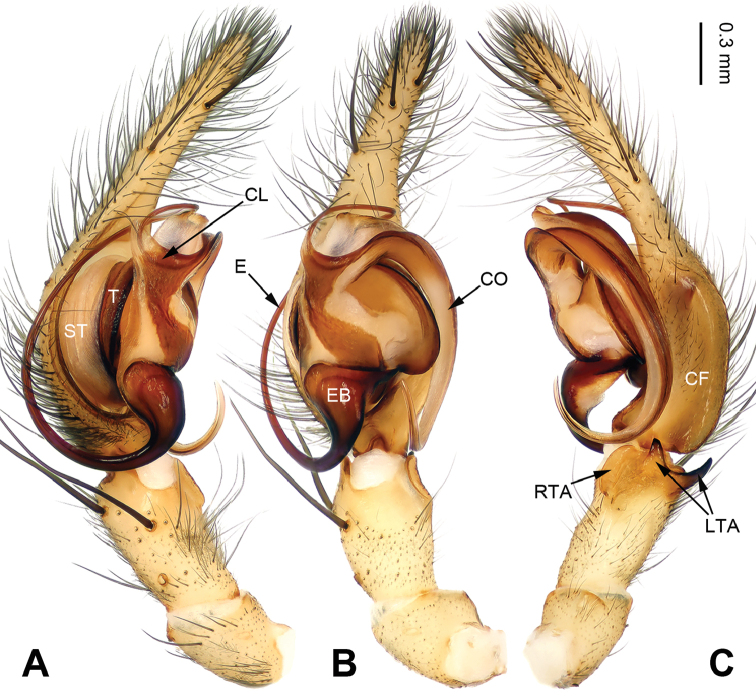
Left palp of *Notiocoelotes maoganensis* sp. n., holotype. **A** Prolateral view **B** Ventral view **C** Retrolateral view. CF = cymbial furrow; CL = conductor lamella; CO = conductor; E = embolus; EB = embolic base; LTA = lateral tibial apophysis; RTA = retroventral tibial apophysis; ST = subtegulum; T = tegulum. Scale bar: Equal for **A, B** and **C**.

**Figure 2.**
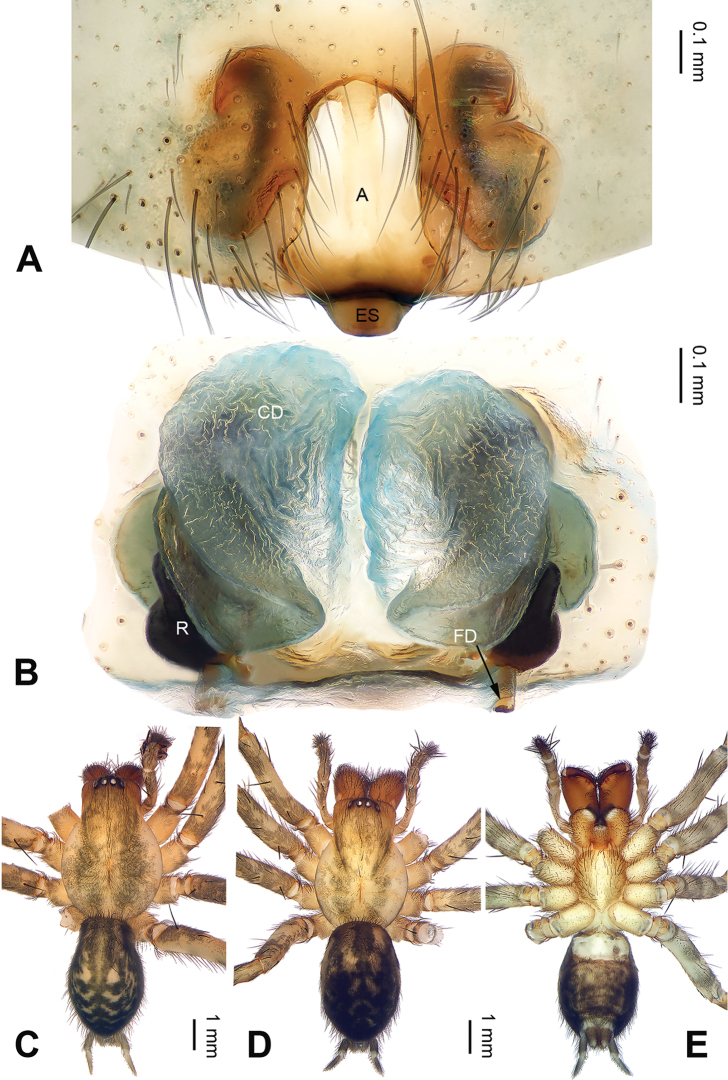
Epigyne and habitus of *Notiocoelotes maoganensis* sp. n. **A** Epigyne, ventral view **B** Vulva, dorsal view **C** Male habitus, dorsal view **D** Female habitus, dorsal view **E** Female habitus, ventral view. A = epigynal atrium; CD = copulatory duct; ES = epigynal scape; FD = fertilization duct; R = receptacle. Scale bars: Equal for **D** and **E**.

The chelicerae of all *Notiocoelotes* have 3 promarginal and 2 retromarginal teeth, while other coelotines usually have 3 or 4 retromarginal teeth. Females of this genus can be separated from other coelotines by the absence of epigynal teeth and the presence of a tongue-shaped epigynal scape (Fig. 2A–B); other coelotines usually have long and broad epigynal teeth. Males can be distinguished from other coelotines by the absence of a patellar apophysis, the presence of a large and strongly bifurcated lateral tibial apophysis and the reduced or invisible median apophysis (Fig. 1); other coelotines usually have a thick patellar apophysis and the special shaped median apophysis.

##### Composition.

Thirteen *Notiocoelotes* species are currently known: *Notiocoelotes laosensis* (♀) from Laos; *Notiocoelotes parvitriangulus* Liu, Li & Pham, 2010 (♀), *Notiocoelotes pseudovietnamensis* Liu, Li & Pham, 2010 (♂♀) and *Notiocoelotes vietnamensis* (♂♀) from Vietnam; *Notiocoelotes sparus* (♂) from Thailand; *Notiocoelotes lingulatus* (♀), *Notiocoelotes membranaceus* (♂), *Notiocoelotes orbiculatus* Liu & Li, 2010 (♂♀), *Notiocoelotes palinitropus* (♂♀), *Notiocoelotes pseudolingulatus* Liu & Li, 2010 (♂♀), and *Notiocoelotes spirellus* Liu & Li, 2010 (♂♀) from Hainan, China (World Spider Catalog 2016), and two new species described in this paper: *Notiocoelotes maoganensis* sp. n. (♂♀), *Notiocoelotes qiongzhongensis* sp. n. (♂♀) from Hainan.

#### Notiocoelotes maoganensis

Taxon classificationAnimaliaAraneaeAgelenidae

Zhao & Li sp. n.

http://zoobank.org/77DC1620-6C90-4167-BA47-CE30A39BF135

Figs 1 , 2 , 7

##### Type material.

**Holotype** ♂: China: Hainan: Baoting County: Maogan Village, Xiananshilin Cave, N18°35'52", E109°25'37", 616 m, 26.VI.2014, F. Li & X. Wang. **Paratype**: 1♀, same data as holotype.

##### Etymology.

The specific name refers to the type locality; adjective.

##### Diagnosis.

The male of *Notiocoelotes maoganensis* sp. n. can be easily distinguished from all other *Notiocoelotes* species, except *Notiocoelotes palinitropus*, by having a semi-circular conductor. From *Notiocoelotes palinitropus*, the male of the new species can be distinguished by the short cymbial furrow about 1/3 of cymbial length (while *Notiocoelotes palinitropus* male has a long cymbial furrow, about 0.5 times as long as cymbial length) (cf. Fig. 1A–C; Zhu and Wang 1994: figs 19–21). The female of *Notiocoelotes maoganensis* sp. n. can be easily distinguished from all the other *Notiocoelotes* species, except *Notiocoelotes palinitropus*, by the almost rectangular atrium. From *Notiocoelotes palinitropus*, the female of the new species can be distinguished by a broad atrium, about two times as long as wide (while *Notiocoelotes palinitropus* female has a narrow atrium, about three times as long as wide) (cf. Fig. 2A–B; Liu and Li 2010: fig. 9B).

##### Description.

**Male (holotype)**: Total length 8.60. Carapace 4.75 long, 3.50 wide. Abdomen 3.85 long, 2.75 wide. Eye sizes and interdistances: AME 0.20, ALE 0.23, PME 0.25, PLE 0.25; AME-AME 0.08, AME-ALE 0.02, PME-PME 0.08, PME-PLE 0.10. Leg measurements: I: 21.15 (5.75, 6.00, 5.50, 3.90); II: 18.00 (5.50, 5.25, 4.50, 2.75); III: 17.00 (5.00, 5.00, 4.75, 2.25); IV: 22.35 (6.25, 6.70, 6.50, 2.90). Carapace yellowish, the radial grooves indistinct, with the nearly lip-shaped dark pattern, sternum yellowish, about almond-shaped. Abdomen brownish, with yellow and transversal spots, nearly oval-shaped. Legs yellowish, with black annulations. Palp: tibia long, about 1/3 of cymbial length; RTA small, 1/3 of tibial length; LTA divided into two parts, most crescent-shaped and about 1/2 length of RTA; conductor long, with one loop; embolus beginning at 6:30 o’clock position, with the triangular base, about 1/3 width of tibia (Fig. 1A–C).

**Female (paratype)**: Total length 8.75. Carapace 4.25 long, 3.50 wide. Abdomen 4.50 long, 3.00 wide. Eye sizes and interdistances: AME 0.20, ALE 0.28, PME 0.25, PLE 0.25; AME-AME 0.05, AME-ALE 0.01, PME-PME 0.08, PME-PLE 0.10. Leg measurements: I: 16.50 (4.75, 5.00, 4.00, 2.75); II: 14.25 (4.25, 4.50, 3.50, 2.00); III: 13.10 (4.00, 4.10, 3.25, 1.75); IV: 17.65 (5.15, 5.75, 4.50, 2.25). Carapace beige, with grey lateral margins; sternum nearly almond-shaped, light brown, with wide yellow median band. Abdomen grey-brown, nearly oval-shaped, with beige herringbone pattern. Legs yellowish, with black annulations. Epigyne: atrium elongated, with distinct septum, about two times as long as wide, posterior broaden; copulatory ducts covering anterior parts of receptacles, about 1.2 times as long as wide; receptacles narrow, about 2.5 times as long as wide; copulatory openings distinct (Fig. 2A–B).

##### Distribution.

Known only from the type locality (Fig. 7).

#### Notiocoelotes membranaceus

Taxon classificationAnimaliaAraneaeAgelenidae

Liu & Li, 2010

Figs 3 , 4 , 7

Notiocoelotes membranaceus Liu & Li, 2010: 33, figs 2A–C, 3A–D (♂).

##### Type material.

**Holotype** ♂: China: Hainan: Qiongzhong County: Mt. Limushan Nature Reserve, 13 August 2007, S. Li, C. Wang, L. Lin & J. Xu leg.

##### Other material examined.

3♀3♂: China: Hainan: Qiongzhong County: Mt. Limushan Nature Reserve, Binlang Lake, N19°11'59", E109°43'45", 576 m, 4.XII.2015, X. Zhang & Z. Chen; 2♀, China: Hainan: Qiongzhong County: Mt. Limushan, N19°10'52", E109°45'19", 962 m, 2.V.2011, Y. Zhou.

##### Diagnosis.

The female of *Notiocoelotes membranaceus* can be distinguished from all the other *Notiocoelotes*, except *Notiocoelotes orbiculatus*, by the almost oval atrium. From *Notiocoelotes orbiculatus*, the new species can be distinguished by the egg-shaped receptacles (while *Notiocoelotes orbiculatus* has globular and widely separated receptacles) (cf. Fig. 4A–B; Liu and Li 2010: fig. 7B).

##### Description.

**Figure 3.**
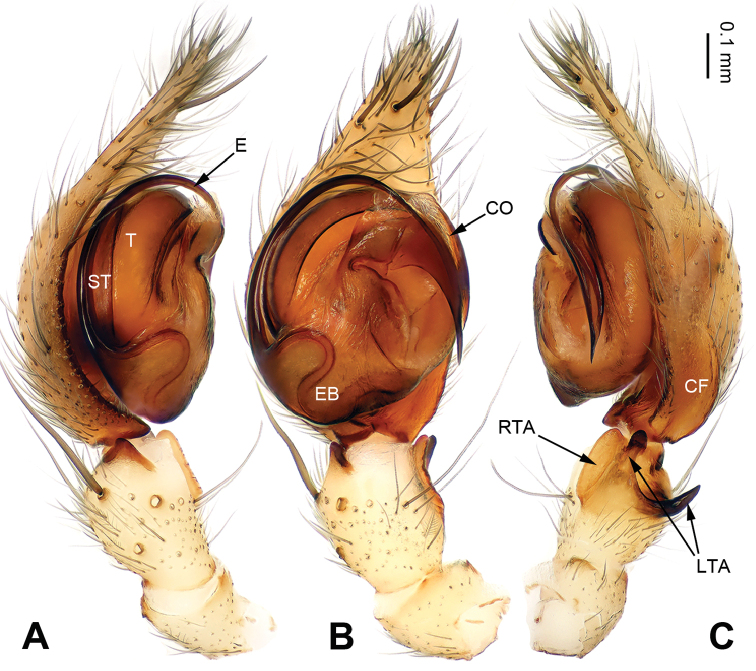
Left palp of *Notiocoelotes membranaceus*, specimen from Hainan. **A** Prolateral view **B** Ventral view **C** Retrolateral view. CF = cymbial furrow; CO = conductor; E = embolus; EB = embolic base; LTA = lateral tibial apophysis; RTA = retroventral tibial apophysis; ST = subtegulum; T = tegulum. Scale bar: Equal for **A, B** and **C**.

**Male**: described in detail by Liu and Li (2010: fig. 2A–C) (Fig. 3A–C).

**Figure 4.**
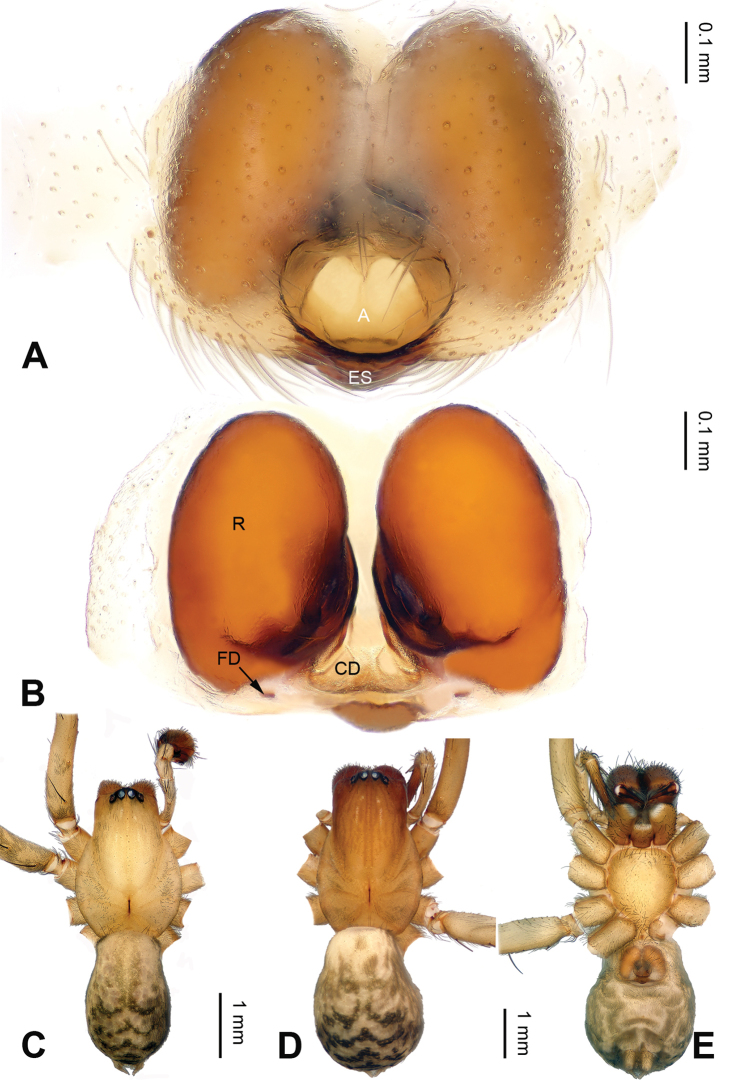
Epigyne and habitus of *Notiocoelotes membranaceus*, specimens from Hainan. **A** Epigyne, ventral view **B** Vulva, dorsal view **C** Male habitus, dorsal view **D** Female habitus, dorsal view **E** Female habitus, ventral view. A = epigynal atrium; CD = copulatory duct; ES = epigynal scape; FD = fertilization duct; R = receptacle. Scale bars: Equal for **D** and **E**.

**Female**: Total length 4.75. Carapace 2.50 long, 1.75 wide. Abdomen 2.25 long, 1.75 wide. Eye sizes and interdistances: AME 0.08, ALE 0.15, PME 0.15, PLE 0.15; AME-AME 0.03, AME-ALE 0.01, PME-PME 0.05, PME-PLE 0.06. Leg measurements: I: 7.00 (2.05, 2.25, 1.60, 1.10); II: 6.10 (1.85, 1.90, 1.55, 0.80); III: 5.50 (1.70, 1.75, 1.30, 0.75); IV: 7.50 (2.25, 2.30, 2.00, 0.95). Carapace yellowish, with black dark lateral margins; sternum yellow, margins darker than median part. Abdomen yellowish-brown, with black and nearly chevrons-shaped stripes, nearly pineapple-shaped. Legs yellowish, with black annulations. Epigyne: atrium semicircular, about 1.5 times as long as wide, with distinct septum; epigynal scape wide; copulatory ducts long, about 0.9 times as long as receptacles, well sclerotized; receptacles long, about 1.5 times as long as wide; copulatory openings distinct (Fig. 4A–B).

##### Distribution.

China (Hainan) (Fig. 7).

##### Remarks.

Female of this species is described for the first time. Although the shape of the palp and epigyne of *Notiocoelotes membranaceus* are a little different from those of the type species of the genus *Notiocoelotes*, the taxonomic placement of this species is supported by the following two features. First, according to the molecular data (our COI sequences, unpublished), *Notiocoelotes membranaceus* is closely related to *Notiocoelotes orbiculatus* and *Notiocoelotes qiongzhongensis* sp. n. Second, the male of *Notiocoelotes membranaceus* has a strongly bifurcated lateral tibial apophysis, characteristic for the males of all *Notiocoelotes* species; the female of *Notiocoelotes membranaceus* has a tongue-shaped epigynal scape, characteristic for the females of all *Notiocoelotes* species.

#### Notiocoelotes qiongzhongensis

Taxon classificationAnimaliaAraneaeAgelenidae

Zhao & Li sp. n.

http://zoobank.org/1AE669ED-AA8C-4EF0-A883-D0E0984D1F55

Figs 5 , 6 , 7

##### Type material.

**Holotype** ♂: China: Hainan: Qiongzhong County: Mt. Limushan, Pine forest, N19°10'53", E109°45'20", 537 m, 2.XII.2015, X. Zhang & Z. Chen. **Paratypes**: 2♀3♂, same data as holotype; 1♀, same area, N19°10'55", E109°45'17", 637 m, 3.V.2011, Y. Zhou.

##### Etymology.

The specific name refers to the type locality; adjective.

##### Diagnosis.

**Figure 5.**
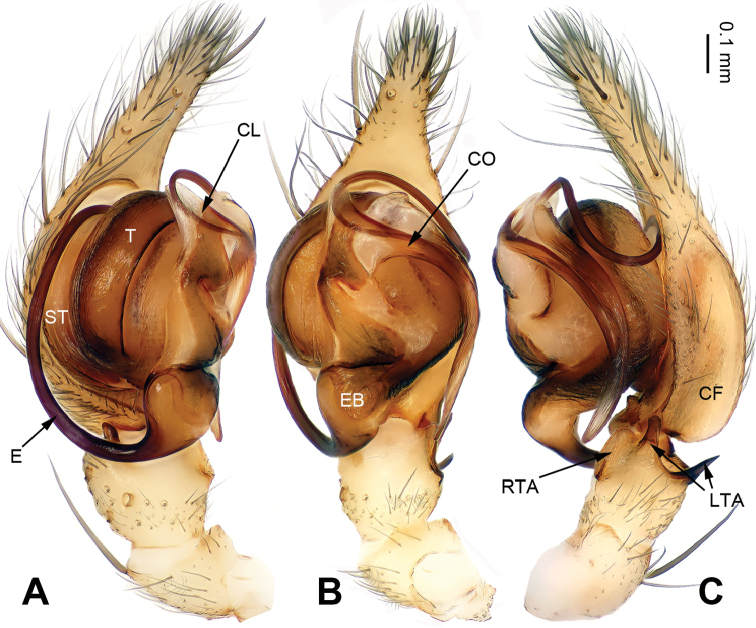
Left palp of *Notiocoelotes qiongzhongensis* sp. n., holotype. **A** Prolateral view **B** Ventral view **C** Retrolateral view. CF = cymbial furrow; CL = conductor lamella; CO = conductor; E = embolus; EB = embolic base; LTA = lateral tibial apophysis; RTA = retroventral tibial apophysis; ST = subtegulum; T = tegulum. Scale bar: Equal for **A, B** and **C**.

**Figure 6.**
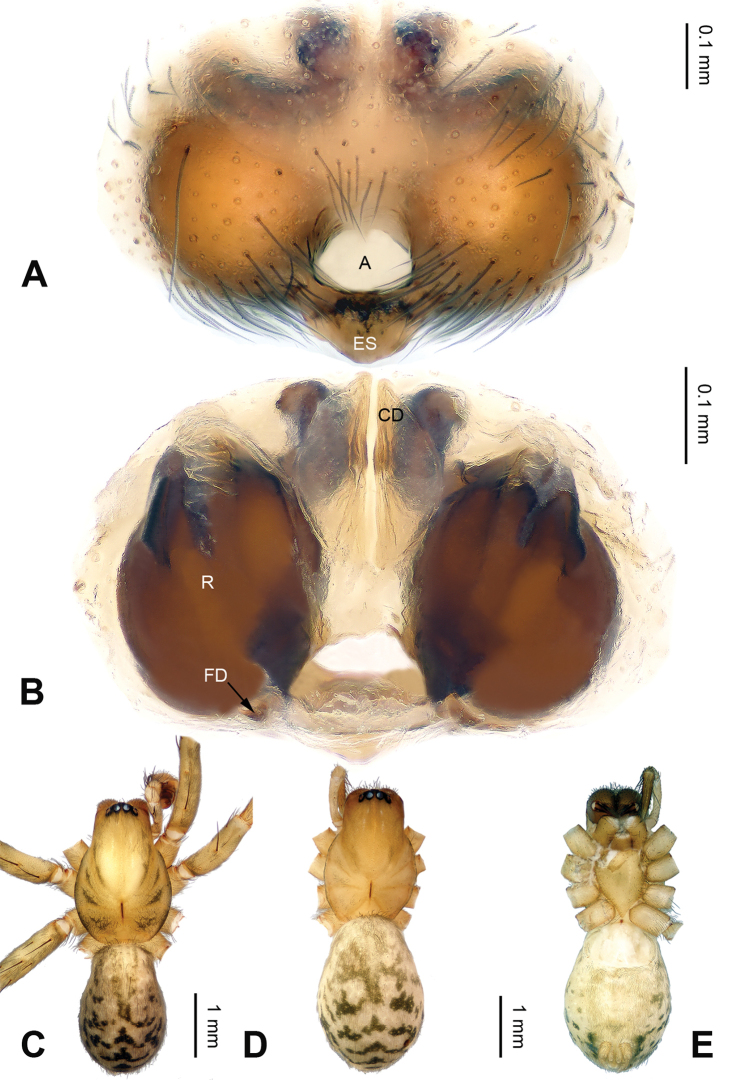
Epigyne and habitus of *Notiocoelotes qiongzhongensis* sp. n. **A** Epigyne, ventral view **B** Vulva, dorsal view **C** Male habitus, dorsal view **D** Female habitus, dorsal view **E** Female habitus, ventral view. A = epigynal atrium; CD = copulatory duct; ES = epigynal scape; FD = fertilization duct; R = receptacle. Scale bars: Equal for **D** and **E**.

The male of *Notiocoelotes qiongzhongensis*, sp. n. can be distinguished from all of the other *Notiocoelotes* species, except *Notiocoelotes pseudolingulatus* and *Notiocoelotes sparus*, by having posteriorly extended conductor and cymbial furrow almost half of cymbial length. From the latter two species, it can be distinguished by the semicircular conductor apex (while *Notiocoelotes pseudolingulatus* has a blunt apex, and *Notiocoelotes sparus* has an acute apex) (cf. Fig. 5A–C; Liu and Li 2010: figs 10–11; Dankittipakul et al. 2005: figs 1–3). The female of *Notiocoelotes qiongzhongensis*, sp. n. can be distinguished from all *Notiocoelotes*, except *Notiocoelotes orbiculatus* and *Notiocoelotes parvitriangulus*, by the rounded receptacles. It can be distinguished from *Notiocoelotes orbiculatus* by the head of receptacles situated on anterior part of receptacles (while the head of receptacles is situated on posterior part of receptacles in *Notiocoelotes orbiculatus*); and it can be distinguished from *Notiocoelotes parvitriangulus* by the nearly square-shaped atrium (while *Notiocoelotes parvitriangulus* has a triangular atrium) (cf. Fig. 6A–B; Liu and Li 2010: fig. 7B; Liu et al. 2010: fig. 78A).

##### Description.

**Male (holotype)**: Total length 4.20. Carapace 2.15 long, 1.60 wide. Abdomen 2.05 long, 1.50 wide. Eye sizes and interdistances: AME 0.05, ALE 0.12, PME 0.15, PLE 0.12; AME-AME 0.04, AME-ALE 0.01, PME-PME 0.04, PME-PLE 0.03. Leg measurements: I: 6.65 (1.95, 2.15, 1.55, 1.00); II: 5.60 (1.75, 1.80, 1.25, 0.80); III: 5.05 (1.50, 1.55, 1.25, 0.75); IV: 7.05 (2.00, 2.25, 1.90, 0.90). Carapace yellowish, with the black and broad radial grooves, with black lateral margins. Abdomen grey, with black spots, nearly eggplant-shaped. Legs yellowish, with black annulations. Palp: tibia short, about 1/4 of cymbial length; RTA about half of tibial length; LTA divided into two parts, almost hook-shaped and subequal the length of RTA; conductor nearly arc-shaped, about 1.5 times as long as tegulum, with two loops; embolus beginning at 7:00 o’clock position, with a nearly chestnut-shaped base, about 1/2 width of tibia (Fig. 5A–C).

**Female (one of paratypes)**: Total length 4.50. Carapace 2.00 long, 1.50 wide. Abdomen 2.50 long, 1.75 wide. Eye sizes and interdistances: AME 0.04, ALE 0.11, PME 0.13, PLE 0.15; AME-AME 0.03, AME-ALE 0.09, PME-PME 0.03, PME-PLE 0.02. Leg measurements: I: 5.15 (1.50, 1.75, 1.15, 0.75); II: 4.50 (1.35, 1.50, 0.95, 0.70); III: 4.05 (1.25, 1.30, 0.90, 0.60); IV: 5.85 (1.75, 1.85, 1.50, 0.75). Carapace yellowish; sternum flavescent. Abdomen beige, with black and wavy stripes, nearly egg-shaped. Legs yellowish, with black annulations. Epigyne: atrium small, almost square-shaped (width=length); receptacles oval, separated by less than 1/2 of their width, about 1.2 times as long as wide; copulatory ducts folded, with two parts, one membranous and another heavily sclerotized cylindrical, almost covered by receptacles; copulatory openings indistinct (Fig. 6A–B).

##### Distribution.

**Figure 7.**
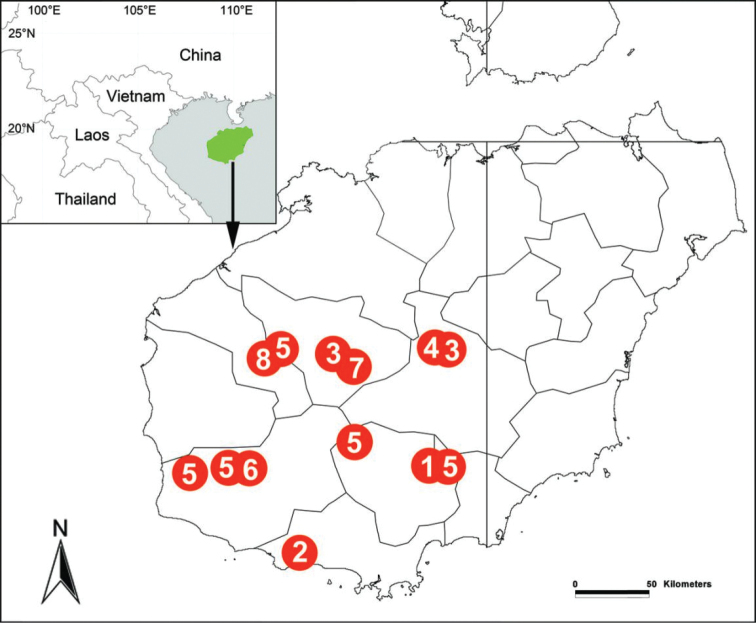
Localities of *Notiocoelotes* species from Hainan. **1** *Notiocoelotes lingulatus* **2** *Notiocoelotes maoganensis* sp. n. **3** *Notiocoelotes membranaceus* **4** *Notiocoelotes orbiculatus* **5** *Notiocoelotes palinitropus* **6** *Notiocoelotes pseudolingulatus* **7** *Notiocoelotes qiongzhongensis* sp. n. **8** *Notiocoelotes spirellus*.

Known only from the type localities (Fig. 7).

## Supplementary Material

XML Treatment for Notiocoelotes

XML Treatment for Notiocoelotes maoganensis

XML Treatment for Notiocoelotes membranaceus

XML Treatment for Notiocoelotes qiongzhongensis
